# Fluorescent polydopamine nanoparticles as a nanosensor for the sequential detection of mercury ions and l-ascorbic acid based on a coordination effect and redox reaction†

**DOI:** 10.1039/d0ra02031a

**Published:** 2020-07-27

**Authors:** Yi-Xuan Yang, Yan-Zhao Fang, Jing-Xuan Tian, Qiang Xiao, Xiang-Juan Kong

**Affiliations:** Jiangxi Key Laboratory of Organic Chemistry, Jiangxi Science and Technology Normal University Nanchang 330013 P. R. China xiaoqiang@tsinghua.org.cn xiangjuankong@163.com +86-791-86422903 +86-791-86422903

## Abstract

Herein, a novel fluorescence nanosensor using intrinsic fluorescent polydopamine nanoparticles (PDA NPs) as an effective signal reporter has been constructed for the simple, rapid and sequential detection of mercury ions (Hg^2+^) and l-ascorbic acid (AA) based on a coordination effect and redox reaction. The fluorescence of the PDA NPs could be specifically quenched by Hg^2+^ through intense coordination effects between the Hg^2+^ and the groups (catechol, amine, ketone and imine) on the surface of the PDA NPs. However, when AA and Hg^2+^ coexisted in solution, the fluorescence of the PDA NPs pronouncedly recovered *via* the redox reaction of Hg^2+^, with it being reduced to Hg^0^ by AA. The fluorescence quenching mechanism of Hg^2+^ towards the PDA NPs and the redox reaction between Hg^2+^ and AA were also fully investigated. The nanosensor exhibited high sensitivity and desirable selectivity for Hg^2+^ and AA detection. Moreover, the strategy was successfully explored in real samples (tap water, lake water and human serum samples) with satisfactory recoveries. The developed nanosensor provides new sights and good inspiration for Hg^2+^ and AA detection under real conditions.

## Abstract 2

A novel fluorescence nanosensor using intrinsic fluorescent polydopamine nanoparticles as an effective signal reporter was constructed for simple, rapid and sequential detection of Hg^2+^ ions and l-ascorbic acid based on a coordination effect and redox reaction.

## Graphical abstract

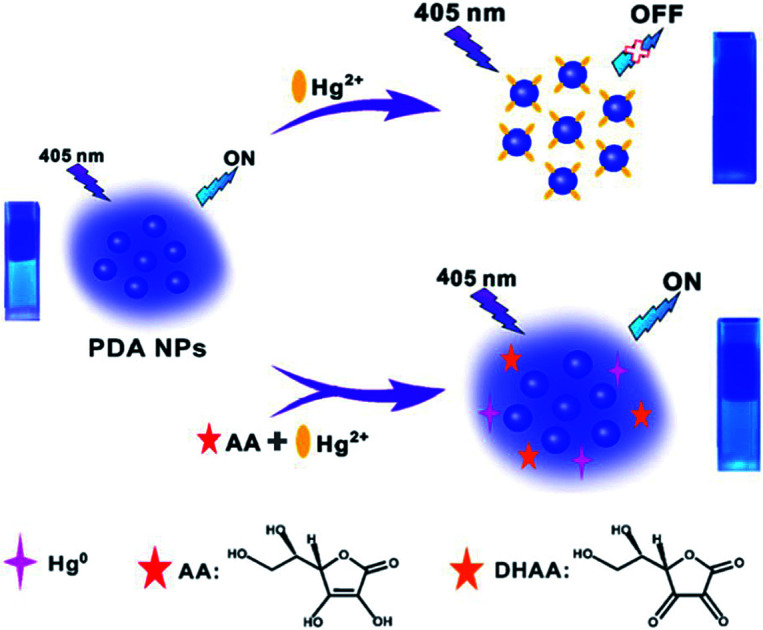

## Introduction

Mercury ions (Hg^2+^), as a highly toxic heavy metal pollutant that abound in the environment, may lead to serious side effects once they enter living organisms, because they are not biodegradable and can accumulate in the body, leading to kidney, eye, respiratory and central nervous system damage.^1,2^l-Ascorbic acid (AA), an essential micronutrient and water-soluble antioxidant, plays a vital role in numerous physiological and biochemical systems, such as oxidative stress reduction, immune cell function and disease prevention in the human body.^3,4^ Moreover, abnormal levels of AA in the human body are closely associated with many diseases, such as rheumatoid arthritis, scurvy, kidney stones, and diarrhea.^5–9^ Therefore, developing simple, rapid, and economical methods for the highly sensitive and selective detection of Hg^2+^ and AA has attracted considerable interest owing to their significant impact on human health.

Up to now, a number of strategies based on electrochemistry,^10,11^ chemiluminescence,^12,13^ chromatography,^14,15^ colorimetry,^16,17^ fluorescence methods and other techniques have been introduced for Hg^2+^ and AA detection.^18–23^ Among them, fluorescence methods have been widely used due to their high sensitivity, rapid response and good reproducibility.^24,25^ Up to now, nanoparticles, including upconversion nanoparticles,^7,26–28^ have emerged for Hg^2+^ and AA detection.^29–34^ Nevertheless, the preparation procedures and reaction conditions of these nanoparticles might be sophisticated and harsh. Thus, developing simple and rapid fluorescence strategies for Hg^2+^ and AA detection is still highly desirable.

More recently, intrinsically fluorescent polydopamine nanoparticles (PDA NPs) have emerged as a novel bioinspired polymer nanoprobe owing to their easy synthesis, good water-solubility and biocompatibility.^35,36^ To the best of our knowledge, intrinsically fluorescent PDA NPs have emerged as a burgeoning field with brilliant prospects in sensing and imaging applications. For instance, Liu *et al.* developed a fluorescent polydopamine dot-based nanosensing strategy for glutamate and Al^3+^ detection in human serum and living cells.^37^ Fu *et al.* reported a facile one-pot method for the synthesis of blue luminescent PDA NPs and developed a nanosystem for the sensitive detection of Fe^3+^.^38^ Chu *et al.*, using MnO_2_ as an oxidant, synthesized the intrinsically fluorescent PDA NPs and established a nanosensor for glutathione detection.^39^ Yang *et al.* extended the application of the intrinsically fluorescent PDA NPs towards the sensitive detection of alkaline phosphatase by employing MnO_2_ nanosheets as an effective nanoquencher.^40^ Jiang *et al.*, using dopamine as a precursor, successfully synthesized fluorescent polydopamine organic nanoparticles, which were further used for developing nucleus-targeting imaging platforms.^41^ The intrinsically fluorescent PDA NPs presented outstanding physicochemical features and displayed promising applications for sensing and imaging. However, the exploitation of intrinsically fluorescent PDA NPs for analytical applications is still at its early stages, and constructing more novel designs and exploring groundbreaking systems for sensing applications is still attractive.

**Scheme 1:**
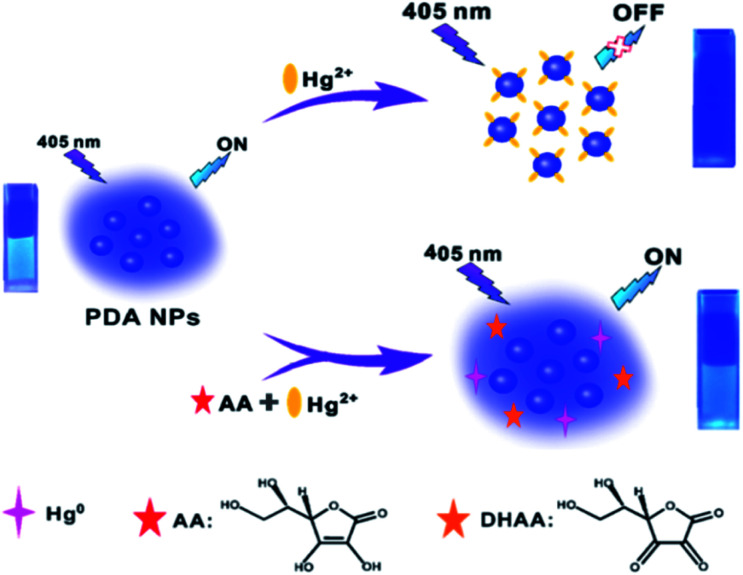
Schematic illustration of the mechanism of the fluorescent PDA NPs-based nanosensor for Hg^2+^ and AA detection.

Herein, we, for the first time, propose a novel fluorescence nanosensor for the sequential detection of Hg^2+^ and AA based on Hg^2+^-induced quenching of intrinsically fluorescent PDA NPs through an intense coordination effect between Hg^2+^ and the groups on the surface of the PDA NPs, and AA-triggered pronounced fluorescence recovery of PDA NPs *via* redox reaction of Hg^2+^, with them being reduced to Hg^0^ by AA. As shown in Scheme 1, the fluorescence of the PDA NPs showed a selective quenching response towards Hg^2+^*via* a coordination effect. Interestingly enough, when AA and Hg^2+^ were added simultaneously into the solution, Hg^2+^ was quickly reduced to Hg^0^, meanwhile, AA, acting as a reductant, was oxidized to dehydroascorbic acid (DHAA),^42^ as shown in the following equation:Hg^2+^ + AA → Hg^0^ + DHAA + 2H^+^And then, as the concentration of AA increased, the fluorescence intensity of the PDA NPs enhanced progressively because of the elimination of the quenching effect. The fluorescent PDA NPs-based nanosensor displayed high sensitivity and selectivity to Hg^2+^ and AA assay. Moreover, the proposed sensor displayed excellent analytical potential for Hg^2+^ and AA detection in real samples, demonstrating promising applications in environmental analysis and biological detection research.

## Experimental

### Reagents and materials

Reduced *N*-ethylmaleimide (NEM) and dopamine hydrochloride were purchased from Sigma-Aldrich. Glutathione (GSH), leucine (Leu), glycine (Gly), cysteine (Cys), homocysteine (Hcy), and AA were purchased from Sangon Biotechnology Company. All the other chemical reagents were of analytical grade. The water used throughout the experiments was obtained from a water purification system with an electric resistance of >18.2 MΩ (Sartorius, Germany). Tap water was obtained from our lab and lake water was obtained from Jingguan Lake at our school. Human serum samples were provided by The First Hospital of Nanchang, Nanchang University (Nanchang, China). All animal procedures were performed in accordance with the Guidelines for the Care and Use of Laboratory Animals of The First Hospital of Nanchang, Nanchang University and approved by the Animal Ethics Committee of The First Hospital of Nanchang, Nanchang University. Our institutional ethics committee specifically approved the absence of informed consent because the data were analyzed anonymously.

Fluorescence measurements were performed in a 96-well black microplate on a Varioskan LUX microplate reader under 405 nm excitation light (Thermo Scientific, USA). The accurate experimental spectroscopic procedure was carried out at a bandwidth of 12 nm. UV-vis absorption measurements were performed in an 8453 UV-vis spectrophotometer (Agilent, USA). Fourier-transform infrared (FT-IR) spectra were measured using a Spectrum Two spectrometer (PerkinElmer, USA). Transmission electron microscopy (TEM) and energy-dispersive X-ray spectroscopy (EDS) images were acquired using a TECNAI G^2^ 20 at an accelerating voltage of 200 kV. X-ray photoelectron spectroscopy (XPS) spectra of the solid samples were measured on an Axis Ultra DLD spectrometer (Shimadzu, Japan).

### Synthesis of fluorescent PDA NPs

The synthesis of fluorescent PDA NPs was carried out according to the reported methods with slight modification.^31,43^ Firstly, the fluorescent PDA NPs were formed by oxidative polymerization *via* the addition of an aqueous solution of dopamine hydrochloride (40 mM, 200 μL) and sodium hydroxide solution (100 mM, 320 μL) into phosphate buffer (PB) (2 mM, pH 7.4, 7.08 mL) under magnetic stirring for 1 h at room temperature. Thereafter, hydrochloric acid (0.2 M, 400 μL) was introduced with stirring for an additional 30 min to sharply reduce the polymerization speed. For purification, the prepared PDA NPs solution was centrifuged for 30 minutes at 12 000 rpm to remove large nonfluorescent PDA NPs. The obtained supernatant was stored at 4 °C for future use.

### Hg^2+^ and AA detection in aqueous solution and real samples

For Hg^2+^ detection, varying concentrations of Hg^2+^ were added into the solution containing 50 μL of PB (pH 7.0, 2 mM) solution and 10 μL as-prepared fluorescent PDA NPs, and enough sterilized water was added into the mixture to adjust the final volume to 100 μL. After mixing, the reaction proceeded at room temperature for 30 min. And then, the solution was subjected to fluorescence measurements.

For AA detection, varying concentrations of AA and 20 μM Hg^2+^ were added into the solution containing 50 μL of PB (pH 7.0, 2 mM) solution and 10 μL of the as-prepared fluorescent PDA NPs, and enough sterilized water was added into the mixture to adjust the final volume to 100 μL. After mixing, the reaction was allowed to proceed at room temperature for 15 min, and was then subjected to fluorescence measurements.

The practicality of the sensor for Hg^2+^ detection was demonstrated in tap water, lake water and diluted human serum samples, and for AA detection was demonstrated in diluted human serum samples. A standard addition recovery method was used for evaluating the feasibility of the fluorescent PDA NPs for Hg^2+^ and AA detection in real samples. In particular, a 20-fold diluted human serum sample was firstly treated with NEM (1.0 mM) and incubated for 30 min to eliminate any interference from sulfhydryl compounds including Cys, Hcy, GSH and Na_2_S (using as H_2_S donor) before testing.

## Results and discussion

### Synthesis and characterization of the fluorescent PDA NPs

**Fig. 1:**
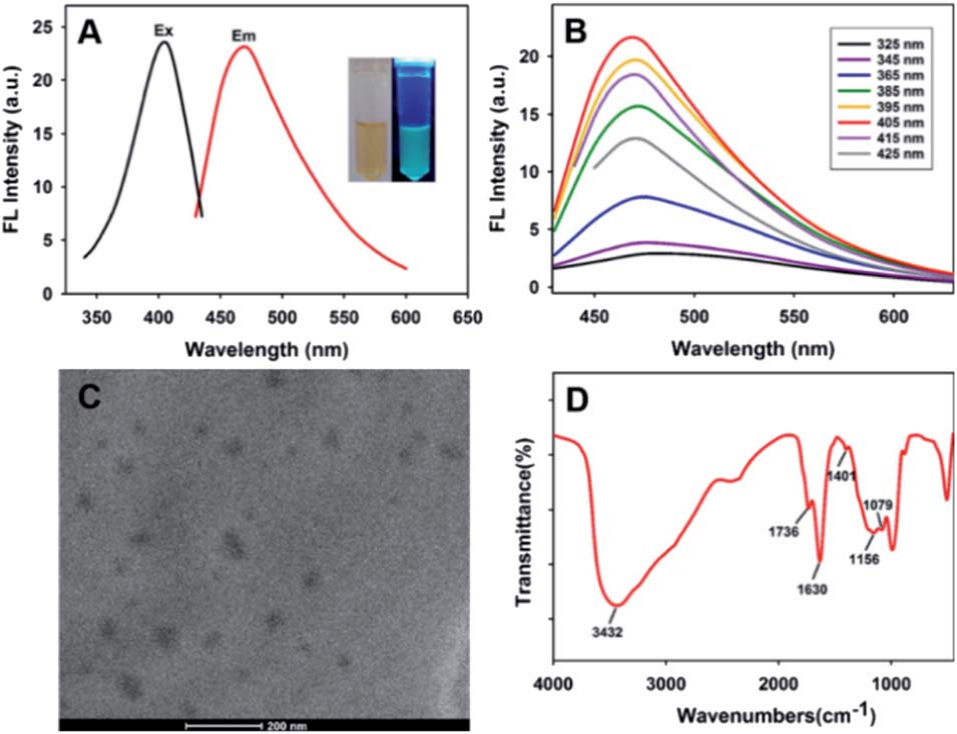
(A) Excitation and emission spectra of the synthesized PDA NPs. The inset shows photographs of the PDA NPs under room light and 365 nm UV light irradiation. (B) Fluorescence emission spectra of the PDA NPs under different excitation wavelengths. (C) TEM images of the PDA NPs. (D) FT-IR results.

The fluorescent PDA NPs were easily synthesized by oxidation and polymerization of dopamine molecules under basic conditions with stirring. The solution was acidified by adding hydrochloric acid to lower the polymerization speed and then fluorescent PDA NPs formed.^43^Fig. 1A showed that the excitation wavelength of the fluorescent PDA NPs was at around 405 nm and the emission intensity reached a maximum at around 468 nm. Apparently, Fig. 1B showed that the emission peak of PDA NPs was excitation-independent, and the excitation wavelength at 405 nm gave the maximum fluorescence output. Fig. S1† shows that the fluorescence signal of the PDA NPs has no obvious decrease within seven days and under continuous irradiation with 405 nm light, revealing the outstanding photostability of the formed PDA NPs and the potential for analysis application.

The size and shape of the formed PDA NPs were characterized by TEM. Fig. 1C shows that the PDA NPs were monodispersed and irregular shaped with diameters in the range of 20–60 nm. Compositional analysis of the formed PDA NPs by EDS exhibited the signals of the C, N and O elements (Fig. S2†). Finally, FT-IR spectroscopy was used to determine the surface chemical groups of the PDA NPs. As shown in Fig. 1D, the strong and broad band located at 3432 cm^−1^ was attributable to the presence of N–H bond and phenolic O–H bond stretching vibrations, the peak appeared at 1401 cm^−1^ corresponded to the bending vibrations of O–H bonds, and the peak located at 1630 cm^−1^ was assigned to the presence of N–H bond bending vibrations and C=C bond stretching vibrations. The peak at 1736 cm^−1^ was ascribed to C=O bonds, the peak at around 1156 cm^−1^ corresponded to the stretching vibration of C–N bonds, and the peak located at around 1079 cm^−1^ was attributable to the stretching vibrations of C–O bonds. The results proved the existence of a number of ketone and hydrophilic groups (catechol, amine, imine) on the surface of the formed PDA NPs, which not only resulted in their excellent water solubility but also provided a platform for metal ion coordination. The water solubility of PDA NPs in water was calculated to be 18.312 mg mL^−1^, which was determined by dissolving excess lyophilized PDA NPs in deionized water. The exhibited water solubility makes it an attractive candidate for sensing applications in solution.

### Feasibility analysis of the sensing system for Hg^2+^ and AA detection

**Fig. 2:**
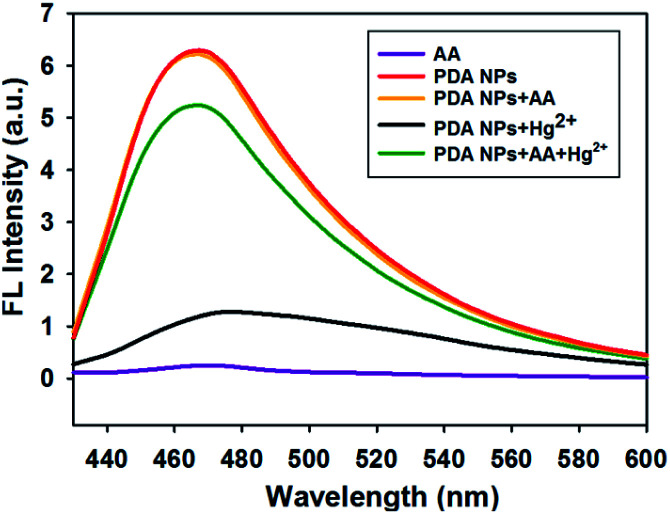
Fluorescence emission spectral responses obtained under different conditions: AA (purple line); PDA NPs (red line); PDA NPs + AA (orange line); PDA NPs + Hg^2+^ (black line); PDA NPs + Hg^2+^ + AA (green line).

The feasibility of the proposed strategy was verified using fluorescence and UV-vis absorption spectroscopic measurements. Fig. 2 shows that the fluorescence signal of the PDA NPs decreased dramatically in the presence of Hg^2+^ (black line). However, the fluorescence signal of the PDA NPs greatly increased upon the addition of AA (green line), which was comparable to the fluorescence intensity of the PDA NPs (red line) or PDA NPs with AA (orange line), suggesting that Hg^2+^ effectively quenched the fluorescence of the PDA NPs and AA successfully recovered the fluorescence signal of the system *via* a redox reaction between the AA and Hg^2+^. UV-vis absorption spectra of the system were also measured, where Fig. S3† shows that the absorption peak shifted remarkably to 286 nm in the presence of Hg^2+^ (blue line). In contrast, when AA and Hg^2+^ coexisted in the solution, the absorption peak was observed at 280 nm (green line), which was the same as the absorption peak of the system without Hg^2+^ (red line). Furthermore, zeta potential measurements revealed the changes in the surface charge of the PDA NPs. Fig. S4† shows that the coordination of Hg^2+^ significantly shifted the zeta potential from −46.17 to −33.63 mV, and after AA was added to the system, the zeta potential shifted to −41.23 mV again. The above results gave clear evidence for the feasibility of the system.

### Mechanism of Hg^2+^-induced fluorescence quenching toward the PDA NPs

**Fig. 3:**
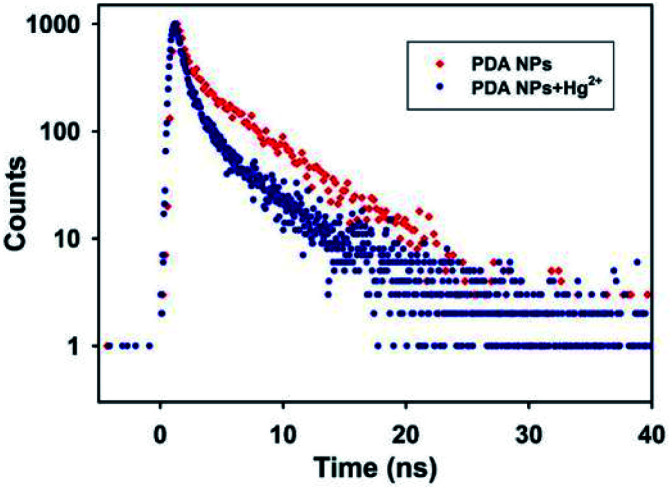
Fluorescence decay curves of the PDA NPs in the absence and presence of Hg^2+^ with the emission monitored at 468 nm and excitation at 375 nm.

The fluorescence quenching process occurs primarily *via* static and dynamic modes. The static mode involves the formation of a nonfluorescent complex *via* a reaction between a fluorescence molecule in the ground state and a quencher, accompanied by fluorescence intensity decreases but the excited-state fluorescence lifetime of the fluorophore does not change. On the contrary, the dynamic mode involves a collision between the excited fluorescence molecule and the quencher, which leads to an excited-state fluorescence lifetime change of the fluorophore.^44–47^ The Hg^2+^-induced fluorescence quenching mechanism toward the PDA NPs was further investigated *via* fluorescence decay curves. Fig. 3 shows that the fluorescence lifetime of PDA NPs in the absence of Hg^2+^ fitted well with a monoexponential function, and the fluorescence life time of the PDA NPs was calculated to be 4.36 ns. Interestingly, in the presence of Hg^2+^, the fluorescence lifetime of the PDA NPs was well fitted with a biexponential function, consisting of a short lifetime component of *τ*_1_ = 0.75 ns (49.17%) and a long lifetime component of *τ*_2_ = 4.49 ns (50.83%). The significantly decreased fluorescence lifetime of *τ*_1_ might result from the occurrence of charge transfer from the PDA NPs to Hg^2+^, which led to the quenching of the fluorescence and proved the existence of dynamic quenching. The unaffected fluorescence life time of *τ*_2_ might be due to the formation of a non-fluorescent complex between Hg^2+^ and the PDA NPs, which confirmed the mode of static quenching. The results validated the fluorescence quenching of the PDA NPs by Hg^2+^ was a combination of static and dynamic quenching processes.

### Demonstration of the redox reaction between AA and Hg^2+^

**Fig. 4:**
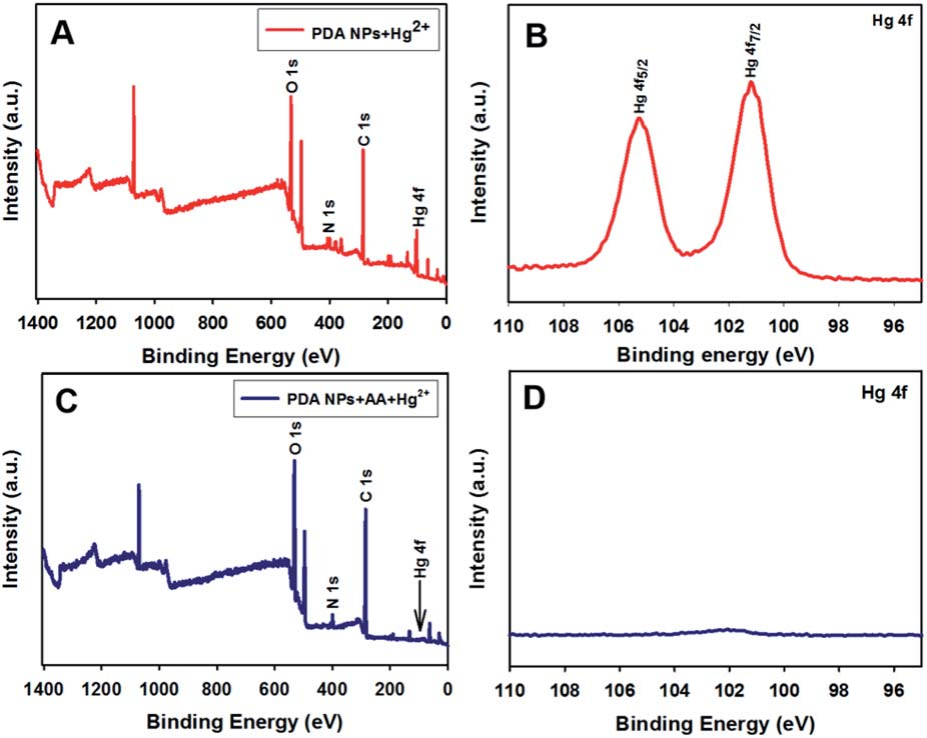
XPS analysis and high-resolution XPS spectra of Hg^2+^ on the surface of the PDA NPs in the absence (A and B) and presence of AA (C and D).

With regard to the demonstration of the redox reaction between the AA and Hg^2+^, XPS spectral analysis was carried out on the surfaces of the PDA NP samples. Fig. 4A shows the survey spectrum that contains C 1s, N 1s, O 1s, and Hg 4f peaks. The high-resolution Hg 4f spectrum (Fig. 4B) shows two peaks located at 101.2 eV for Hg 4f_7/2_ and 105.2 eV for Hg 4f_5/2_. The location of the peaks is characteristic of Hg^2+^.^48^ In contrast, the presence of the AA in the system resulted in Hg^2+^ reduction to Hg^0^, which was not coordinated by the PDA NPs. When analyzing PDA NPs treated with Hg^2+^ in the presence of AA, the peaks for Hg 4f disappeared in both the survey (Fig. 4C) and high-resolution (Fig. 4D) Hg 4f spectra. The results indicated the occurrence of the redox reaction of Hg^2+^ being reduced to Hg^0^ by AA, as was previously reported.^49^

### Optimization of the experimental conditions

Before investigating the detection behavior of the sensing system, several experimental parameters, including the acidity (pH) of the solution and reaction time for Hg^2+^ detection, and the adding order of the chemicals and reaction time for AA detection were investigated to systematically optimize the experimental conditions of the detection method.

#### Optimization of the experimental conditions for Hg^2+^ detection

The influence of the solution pH was first demonstrated because the protonation and deprotonation of the amine, imine, and phenol groups on the surface of the PDA NPs were dramatically changed according to the variation of the solution pH, thus resulting in different quenching responses of the PDA NPs towards Hg^2+^. Fig. S5A† shows that the fluorescence intensity increased when the solution pH was increased from 5.0 to 6.5, and remained stable in the pH range of 7.0–9.0. However, once Hg^2+^ was added, the fluorescence intensity decreased obviously in the pH range of 4.0–7.0, and then gradually increased when the pH of the solution was increased from 7.5 to 9.0, as shown in Fig. S5B.† Therefore, a solution pH of 7.0 was selected for further study.

To realize the fast and highly sensitive detection of Hg^2+^, the reaction time of the system for Hg^2+^ detection was investigated. Fig. S5C† shows that the fluorescence intensity of the PDA NPs decreased sharply when the reaction time was varied from 0 to 30 min, and then remained unchanged after 30 min. Thus, 30 min was used for the subsequent experiments.

#### Optimization of the experimental conditions for AA detection

Most notably, the increase in the fluorescence intensity of the sensing system for AA detection was dependent on order that the chemicals were added into the solution. As shown in Fig. S6A,† if the PDA NPs and Hg^2+^ were first added to the solution and it was kept at room temperature for 30 min the PDA NPs exhibited an inconspicuous fluorescence signal after the addition of the AA even after it was reacted at room temperature for 45 min (orange line, group A), which might be ascribed to the strong coordination reaction between the Hg^2+^ and the groups on the surface of the PDA NPs that interfere with the redox reaction between the AA and Hg^2+^. By contrast, in group B, the PDA NPs and Hg^2+^ were first added into the solution followed by the addition of the AA immediately, in which the PDA NPs displayed significant fluorescence signal enhancement (blue line) after 30 min of co-incubation. In particular, in the group C adding of the chemicals in the order of PDA NPs, AA and Hg^2+^, desirable fluorescence signal response (red line) was observed after 30 min of co-incubation. Hence, to realize highly sensitive AA detection, the addition order of group C was chosen for further study.

To realize the rapid detection of AA, the reaction time of the system for AA detection was also investigated. Fig. S6B† shows that the fluorescence intensity of the PDA NPs increased rapidly when the reaction time varied from 0 to 15 min, and then leveled off at 15 min. Thus, 15 min was used for subsequent experiments.

### Detection procedure

**Fig. 5:**
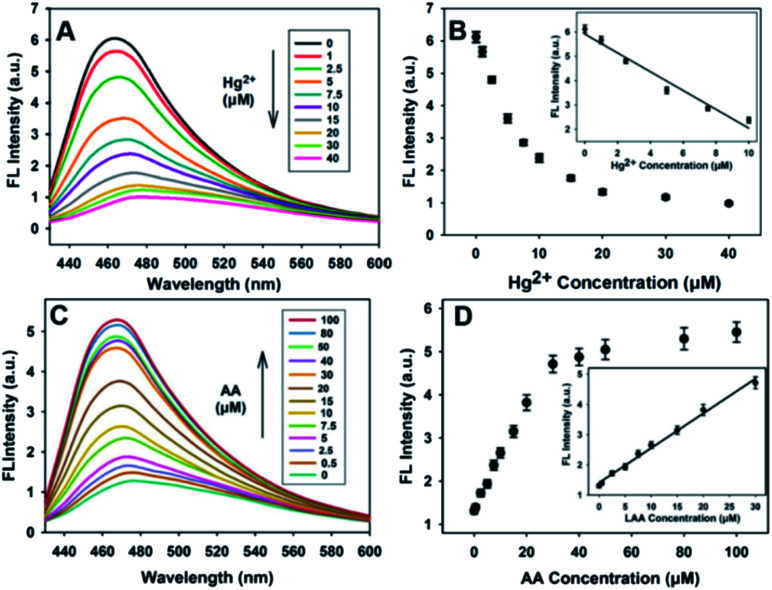
(A) The fluorescence spectral responses of the PDA NPs towards a series of various Hg^2+^ concentrations. (B) Relationship between the fluorescence intensity of the PDA NPs at 468 nm and the Hg^2+^ concentrations (0–40 μM). The inset shows the linear range from 0 to 10 μM. (C) Fluorescence profiles of the PDA NPs in the presence of various AA concentrations (0–100 μM). (D) The corresponding fluorescence spectral responses *versus* AA concentrations. The inset shows the linear range from 0 to 30 μM. The error bars represent the standard deviation of three repeated experiments.

The fluorescence responses of the nanosensor to Hg^2+^ and AA in buffer solutions were investigated under the optimal experimental conditions. Fig. 5A shows that the fluorescence intensity of the PDA NPs decreased sharply when the concentration of Hg^2+^ increased from 0 to 10 μM, and then became sluggish even when the concentration of Hg^2+^ was increased beyond 10 μM. Good linearity between the relative fluorescence intensity and the concentration of Hg^2+^ (0–10 μM) was obtained (*R*^2^ = 0.9801), as shown in Fig. 5B, and the detection limit was found to be 0.19 μM based on the 3*σ* rule. Fig. 5C shows the fluorescence spectral responses of the sensor with different concentrations of AA. As expected, the fluorescence intensity correlated well upon increasing the concentration of AA (0–100 μM). The fluorescence intensity apparently increased, which was as a result of the redox reaction between the AA and Hg^2+^. Fig. 5D shows that there is good linearity between the relative fluorescence intensity and the concentration of AA up to a concentration of 30 μM was reached (*R*^2^ = 0.9934). The detection limit was found to be 0.4 μM based on the 3*σ* rule. The detection results for Hg^2+^ and AA were better than or comparable to the results in previous reports (Table S1†), demonstrating that the developed sensor has many obvious advantages, for instance, simplicity, rapid implementation and high sensitivity for Hg^2+^ and AA detection.

### Selectivity of the proposed sensor

**Fig. 6:**
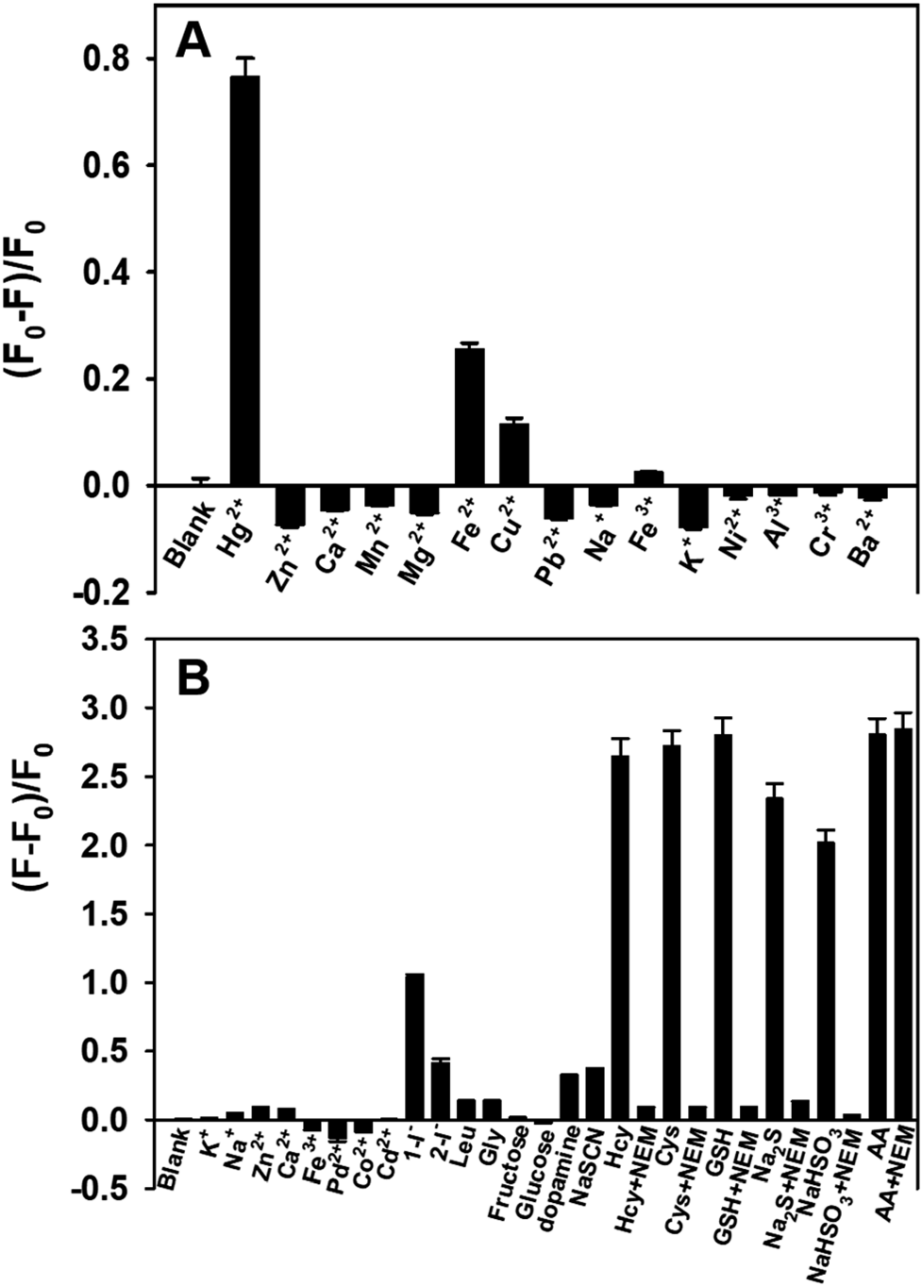
(A) Selectivity of the sensor for Hg^2+^ detection. The concentrations of Hg^2+^, Fe^2+^, Cu^2+^ and Al^3+^ were 20 μM, the concentrations of the other metal ions were 100 μM. *F*_0_ and *F* are the fluorescence intensities of the PDA NPs at 468 nm in the absence and presence of metal ions, respectively. (B) Selectivity of the sensor for AA detection. The concentrations of Pd^2+^, I^−^, AA, dopamine, NaSCN, Hcy, Cys, GSH and Na_2_S were 100 μM, the concentrations of other interfering species were 1 mM and the NEM concentration was 0.6 mM. 1-I^−^ represents that I^−^ was added before Hg^2+^, 2-I^−^ represents that I^−^ was added after Hg^2+^. *F*_0_ and *F* are the fluorescence intensities of the PDA NPs in the absence and presence of interfering species or AA, respectively. The error bars represent the standard deviation of three repeated experiments.

The selectivity of the proposed method for Hg^2+^ and AA detection was evaluated in the presence of a variety of possible interferences. Fig. 6A shows that when the fluorescent PDA NPs were incubated with Hg^2+^, Cu^2+^ and Fe^2+^, the fluorescence intensity diminished compared with the other metal ions. While Fe^2+^ and Cu^2+^ ions influence the mercury detection of the PDA NPs, these inputs can in the future be separated using a logic gate concept.^50,51^ Also, Fig. 6B shows that significant fluorescence intensity enhancements were conspicuously observed in the presence of Hcy, Cys, GSH, NaHSO_3_, Na_2_S and AA, while other interfering species showed slight changes, even at higher concentration. Fortunately, the interferences from Hcy, Cys, GSH, NaHSO_3_ and Na_2_S could be effectively eliminated by NEM, a specific scavenger that forms stable thioether bonds with sulfhydryl compounds, whereas the fluorescence intensity of the PDA NPs for AA detection was essentially unchanged after the introduction of NEM. Notably, I^−^ caused a slight fluorescence increase when the concentration was 100 μM, but it could be minimized by changing order in which the chemicals were added. In addition, the fluorescence signals caused by dopamine and NaSCN were nearly negligible when the concentrations of them were the same as AA. The results fully indicated the high selectivity of the nanosensor for Hg^2+^ and AA detection.

### Analysis of Hg^2+^ and AA in real samples

The practical utility of the nanosensor for Hg^2+^ and AA detection in complex media was demonstrated in real samples (tap water, lake water and diluted human serum samples). With four different concentrations of Hg^2+^ spiked into tap water, lake water and the diluted human serum samples respectively, recoveries of between 93.4% and 107.5% were obtained with RSDs below 6.8% (Table S2†). Diluted human serum samples were selected as biological media to assess the practicality of the sensor for AA detection. Fig. S7† shows that the fluorescence intensity of the PDA NPs enhanced progressively as the AA concentration increased and a linear response to AA ranging from 0–20 μM was observed. In terms of the calibration curve of the serum, four serum samples with different concentrations of AA were prepared and measured, and satisfactory recoveries of between 95.6% and 102.8% were obtained with RSDs of around 5% (Table S3†). The results demonstrated that the developed strategy has potential practicability for Hg^2+^ and AA detection in real samples.

## Conclusions

In summary, the rationale of a simple, rapid and sensitive nanosensor using fluorescent PDA NPs for the sequential detection of Hg^2+^ and AA has been demonstrated. The sensor was constructed mainly based on a Hg^2+^-induced fluorescence quenching effect toward PDA NPs through an intense coordination interaction between Hg^2+^ and the groups on the surface of the PDA NPs, and AA-triggered induced fluorescence recovery of the PDA NPs *via* a redox reaction between Hg^2+^ and AA. By employing the fluorescent PDA NPs as signal readout molecules, the nanosensor exhibited high sensitivity and desirable selectivity. Moreover, the method showed potential applications for Hg^2+^ and AA detection, with satisfactory recoveries in real samples, which may provide new insight for applications in environmental analysis and biological detection diagnosis.

## Conflicts of interest

There are no conflicts to declare.

## Supplementary Material

RA-010-D0RA02031A-s001
