# Supplementary material for: Fluorescent polydopamine nanoparticles as a nanosensor for the sequential detection of mercury ions and l-ascorbic acid based on a coordination effect and redox reaction
Source: RSC Adv. 2020 Jul 27;10(47):28164–70. doi: 10.1039/d0ra02031a (PMC9055638; doi:10.1039/d0ra02031a)
Supplement: RA-010-D0RA02031A-s001 [file RA-010-D0RA02031A-s001.pdf]

Electronic Supplementary Material (ESI) for RSC Advances.
This journal is © The Royal Society of Chemistry 2020

Electronic Supplementary Material (ESI) for Analyst
This journal is © The Royal Society of Chemistry 2013

Supporting Information

Fluorescent polydopamine nanoparticles as a nanosensor for sequential detection of mercury ion and L-ascorbic acid based on coordination effect and redox reaction

Yi-Xuan Yang[1], Yan-Zhao Fang[1], Jing-Xuan Tian, Qiang Xiao* and Xiang-Juan Kong*

*Jiangxi Key Laboratory of Organic Chemistry, Jiangxi Science and Technology Normal University, Nanchang 330013, P. R. China**

Email:xiaoqiang@tsinghua.org.cn; xiangjuankong@163.com

[1] These authors contributed equally to this work.

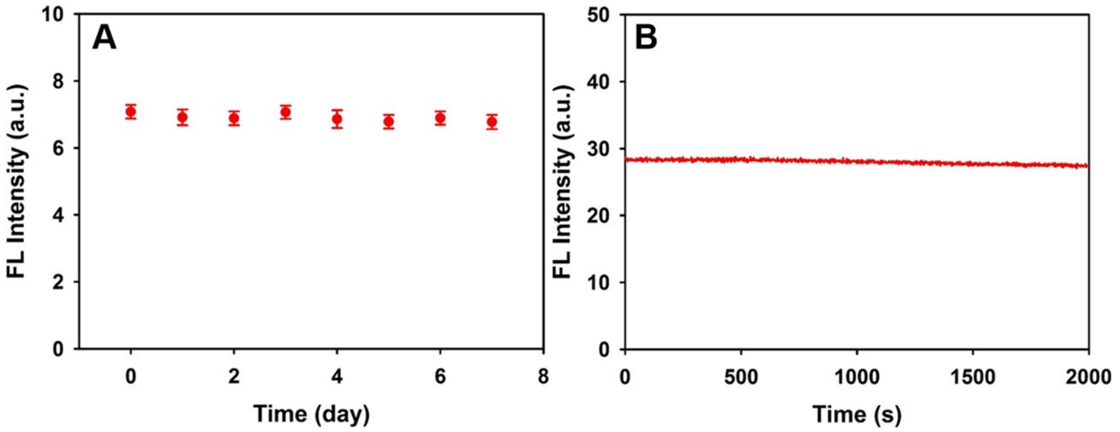

Fig. S1 Fluorescence stability of the as-prepared PDA NPs in solutions within different days (A, 50 μL PB (pH 7.0, 2 mM) solution+10 μL fluorescent PDA NPs+40 μLsterilized water) and under continuous irradiation with 405nm excitation light (B, 50 μL PB (pH 7.0, 2 mM) solution +50 μL fluorescent PDA NPs). Error bars represent standard deviation of three repetitive experiments.

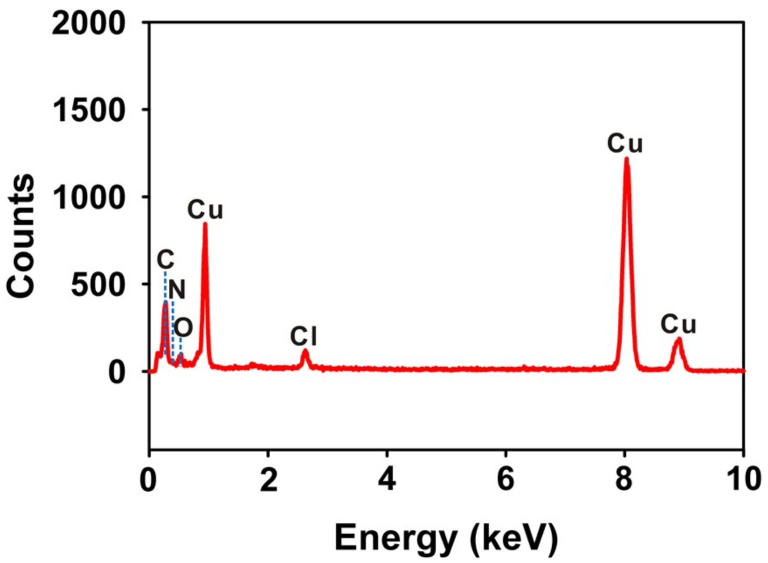

Fig. S2 EDS spectrum of the PDA NPs, indicating the presence of elemental C, N, and O. Note that the strong signal of Cu comes from the copper TEM grid and the signal of Cl come from hydrochloric acid.

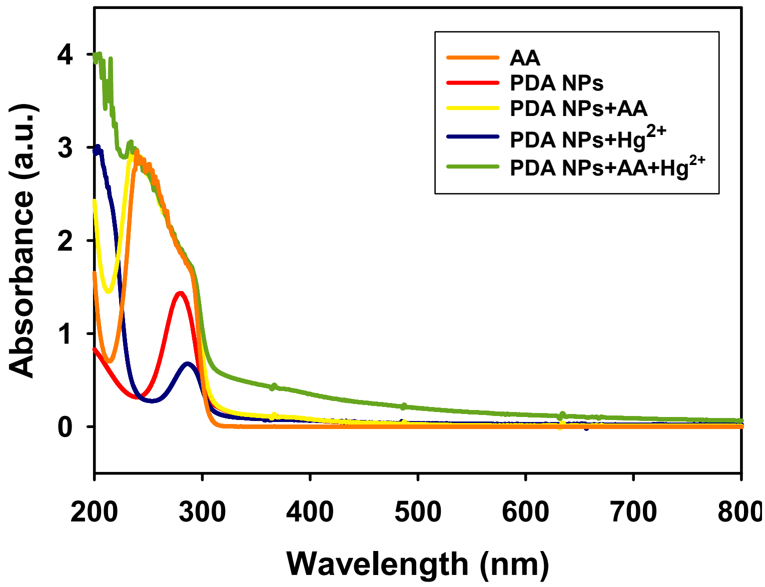

Fig. S3 UV-Vis absorption spectrum responses of the nanoprobe in the presence of PDA NPs (red line, 50 μL PB (pH 7.0, 2 mM) solution+40 μL fluorescent PDA NPs+10 μL sterilized water), PDA NPs+Hg^{2+} (blue line, 50 μL PB (pH 7.0, 2 mM) solution+40 μL fluorescent PDA NPs+5 μL Hg^{2+} (20 mM)+5 μL sterilized water), PDA NPs+AA (yellow line, 50 μL PB (pH 7.0, 2 mM) solution+40 μL fluorescent PDA NPs+5 μL AA (40 mM)+5 μL sterilized water), PDA NPs+AA+ Hg^{2+} (green line, 50 μL PB (pH 7.0, 2 mM) solution+40 μL fluorescent PDA NPs+5 μL Hg^{2+} (20 mM)+5 μL AA (40 mM)) and AA (orange line, 50 μL PB (pH 7.0, 2 mM) solution+5 μL AA (40 mM)+45 μL sterilized water).

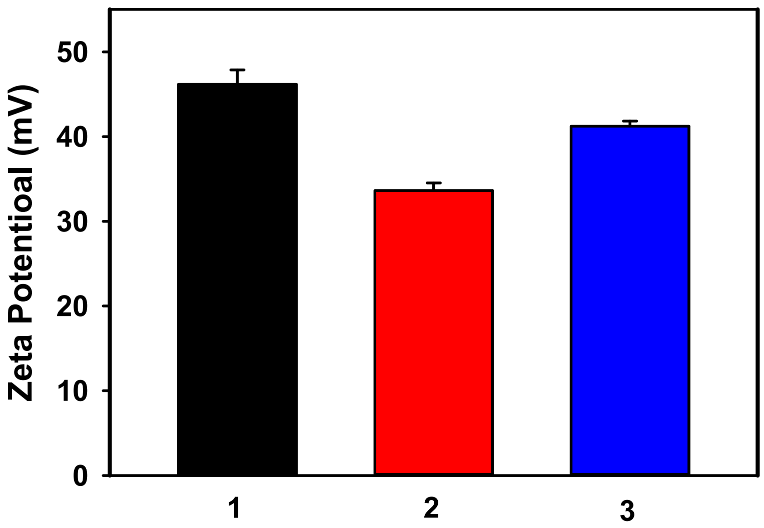

Fig. S4 Zeta potential analysis of samples under different conditions: 1, PDA NPs; 2, PDA NPs+Hg^{2+}; 3, PDA NPs+AA+Hg^{2+}.

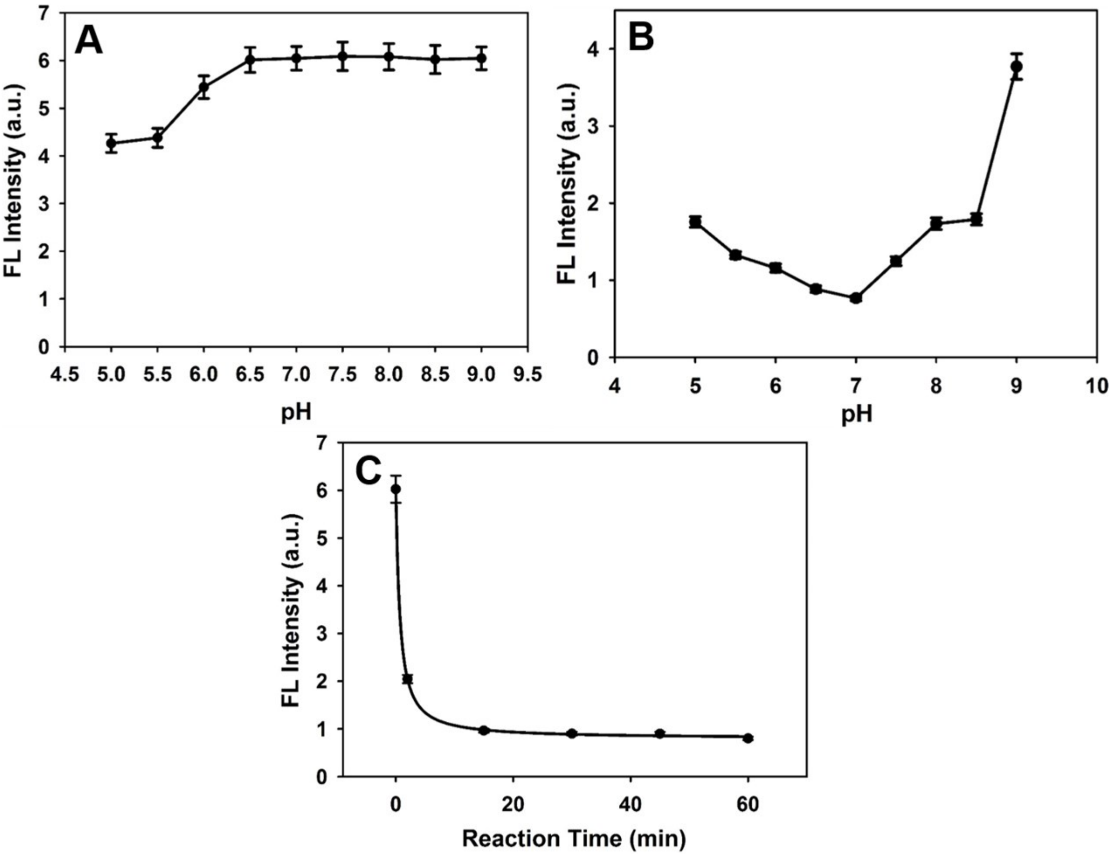

Fig. S5 Optimization of the experimental conditions for Hg^{2+} detection. (A) The effect of the solution pH on the fluorescence intensity of PDA NPs. (B) The effect of the solution pH on the coordination reaction between Hg^{2+} and PDA NPs. (C) Variance of the fluorescence intensity with reaction time of the system for Hg^{2+} detection. Error bars represent the standard deviation of three experiments.

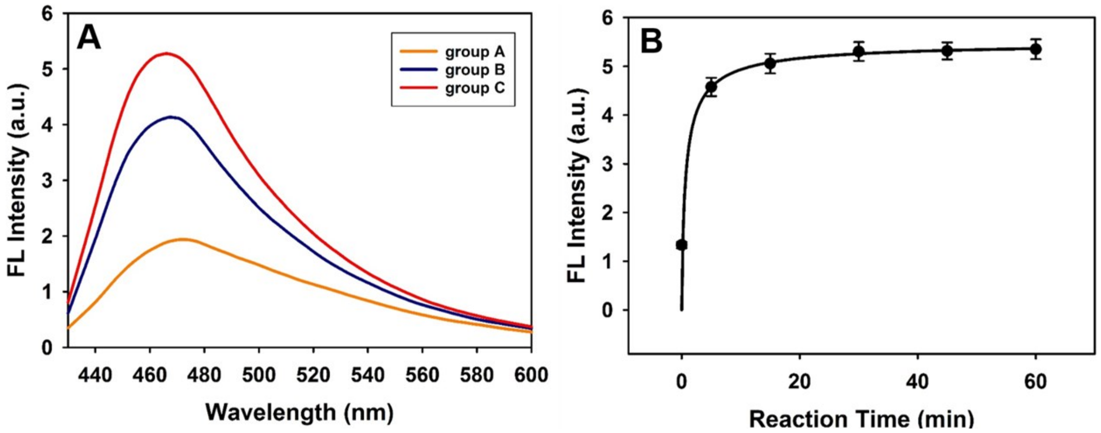

Fig. S6 Optimization of the experimental conditions for AA detection. (A) Variance of the fluorescence intensity with the adding order of AA. Group A: firstly adding PDA NPs and Hg^{2+} for reaction 30 min and then adding AA followed by incubation for 45 min); group B: adding samples according to the order of PDA NPs, Hg^{2+} and AA, and co-incubation for 30 min; group C: adding samples according to the order of PDA NPs, AA and Hg^{2+}, and co-incubation for 30 min. (B) Variance of the fluorescence intensity with the reaction time of the system for AA detection. Error bars represent the standard deviation of three experiments.

Table S1. Comparison of different analytical methods for AA detection sensitivity by our strategy and those previously reported in literature

Method	Analyte	Detection Limit (μM)	Strategy	Ref.
Fluorescence	Hg^{2+}	0.23	A "turn-off" fluorescent probe based on nitrogen-doped carbon quantum dots for detection of Hg^{2+} ions	1
Fluorescence	Hg^{2+}	0.15	Label-free turn-on fluorescent detection of melamine based on the anti-quenching ability of Hg^{2+} to gold nanoclusters	2
Colorimetry	Hg^{2+}	2.2	Highly selective Hg^{2+} colorimetric sensor using green synthesized and unmodified silver nanoparticles	3
Fluorescence	Hg^{2+}	0.39	A dual sensor based on cinamaldehyde and pyrimidine selective for Hg^{2+} and cysteine detection	4
Fluorescence	Hg^{2+}	0.19	Fluorescent PDA NPs serving as signal indicator based on Hg^{2+}-induced fluorescence quenching and AA-triggered fluorescence recovery	This work
Fluorescence	AA	3	By combining the reduction reaction mediated formation of Ag NPs and the selective recognition reaction between Ag^{+} and CdTe QDs	5
Colorimetry	AA	25	Colorimetric detection based on Cu^{2+} ions were able to induce Au NRs to form high-index {200} facet	6
Colrimetry	AA	90	Colorimetric method based on molybdenum oxide nanosheets and AA	7
Electrochemistry	AA	0.51	Nitrogen-doped graphene-like mesoporous nanosheets from the niomass waste of okara for the amperometric detection of vitamin C	8
Electrochemistry	AA	0.5	Yttrium hexacyanoferrate microflowers on freestanding three dimensional graphene substrates	9
Fluorescence	AA	0.4	Fluorescent PDA NPs serving as signal indicator based on Hg^{2+}-induced fluorescence quenching and AA-triggered fluorescence recovery	This work

Table S2. Recovery detection of Hg^{2+} in real samples

		added(μM)	found(μM)	recovery	RSD(n=3)
Tap	1	2.5	2.40	96.0%	5.9%
water	2	5.0	4.67	93.4%	5.0%
	3	7.5	8.06	107.5%	2.3%
	4	10.0	9.64	96.4%	6.2%
Lake	5	2.5	2.37	94.8%	5.7%
water	6	5.0	4.75	95.0%	6.8%
	7	7.5	7.47	99.6%	5.5%
	8	10.0	9.77	97.7%	4.1%
0.5%	9	2.5	2.52	100.8%	3.7%
human	10	5.0	5.12	102.4%	1.0%
serum	11	7.5	7.55	100.7%	6.5%
	12	10.0	9.86	98.6%	6.4%

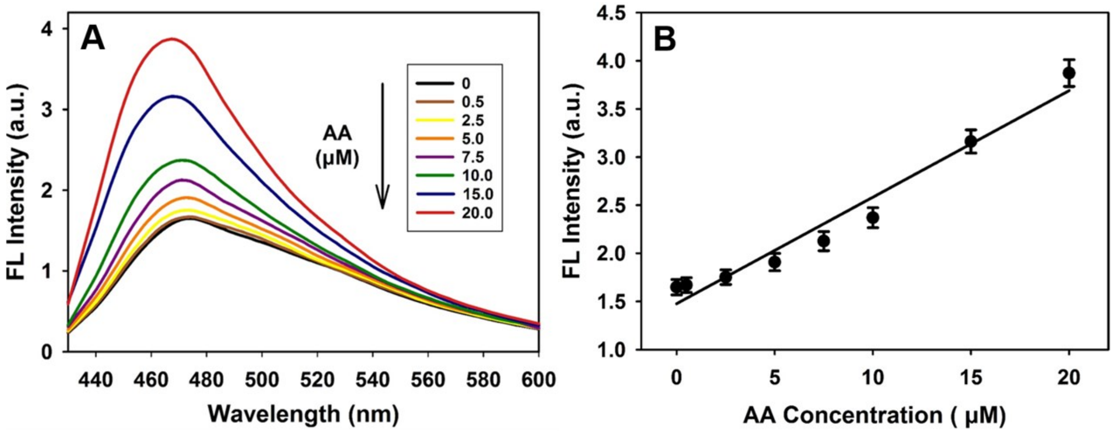

Fig. S7 The calibration curve for AA detection in diluted serum. (A) Fluorescence spectrum responses of the PDA NPs as a function of AA (0-20 μM) in human serum. (B) Plot of the fluorescence intensity values against AA concentration. Error bars represent the standard deviation of three experiments.

Table S3. Recovery detection of AA in 0.5% human serum samples

	added(μM)	found(μM)	recovery	RSD(n=3)
1	5	4.79	95.8%	4.4%
2	7.5	7.71	102.8	5.1%
3	10	9.73	97.3%	3.3%
4	15	14.34	95.6%	6.0%

References:

1. R. Zhang and W. Chen, *Biosens. Bioelectron.*, 2014, **55**, 83-90.

2. H. Dai, Y. Shi, Y. Wang, Y. Sun, J. Hu, P. Ni, and L. Zhuang, *Biosens. Bioelectron.*, 2014, **53**, 76-81.

3. K. Farhadi, M. Forough, R. Molaei, S. Hajizadeh and A. Rafipour, *Sens. Actuators B: Chem.*, 2012, **161**, 880-885.

4. J. M.Jung, C. Kim and R. G. Harrison, *Sens. Actuators B: Chem.*, 2018, **255**, 2756-2763.

5. P. Chen, S. Yan, E. Sawyer, B. Ying, X. Wei, Z. Wu and J. Geng, *Analyst*, 2019, **144**, 1147-1152.

6. S. Xu, X. Dong, S. Chen, Y. Zhao, G. Shan, Y. Sun, Y. Chen and Y. Liu, *Sens. Actuators B: Chem.*, 2019, **281**, 375-382.

7. R. Li, H. An, W. Huang and Y. He, *Sens. Actuators B: Chem.*, 2018, **259**, 59-63.

8. T. Sha, J. Liu, M. Sun, L. Li, J. Bai, Z. Hu and M. Zhou, *Talanta*, 2019, **200**, 300-306.

9. A. Hatamie, R. Rahmati, E. Rezvani, S. Angizi and A. Simchi, *ACS Appl. Nano Mater.*, 2019, **2**, 2212-2221.
